# Quantitative and Qualitative Airborne Mycobiota Surveillance in High-Risk Hospital Environment

**DOI:** 10.3390/microorganisms11041031

**Published:** 2023-04-14

**Authors:** Aleksandra Górzyńska, Aneta Grzech, Paulina Mierzwiak, Marek Ussowicz, Monika Biernat, Urszula Nawrot

**Affiliations:** 1 Department of Pharmaceutical Microbiology and Parasitology, Faculty of Pharmacy, Wroclaw Medical University, Borowska 213a, 50-556 Wroclaw, Polandanetanowak1989@gmail.com (A.G.);; 2 Department of Paediatric Bone Marrow Transplantation, Oncology and Haematology, Wroclaw Medical University, Supraregional Centre of Paediatric Oncology “Cape of Hope”, 50-556 Wrocław, Poland; marek.ussowicz@umw.edu.pl; 3 Department of Haematology, Blood Neoplasms and Bone Marrow Transplantation, Wroclaw Medical University, 50-367 Wroclaw, Poland; monika.biernat@umw.edu.pl

**Keywords:** hospital indoor air, air sampling, *Aspergillus*, *Fusarium*, *Penicillium*, triazole

## Abstract

(1) Background: The primary aim of the presented study was to assess the prevalence of fungi in the indoor air of selected hospital wards, and the additional goal was to evaluate the susceptibility of cultured isolates of *Aspergillus fumigatus* to triazoles. (2) Methods: Three hematology departments and a hospital for lung diseases were surveyed in 2015 and/or 2019. Air samples were taken with a MicroBio MB1 air sampler on Sabouraud agar. The susceptibility of *Aspergillus fumigatus* isolates to voriconazole, posaconazole and itraconazole was tested with a microdilution method, according to EUCAST. (3) Results: The amount of fungi cultured from rooms equipped with sterile air circulation, as well as flow devices for air disinfection, was significantly lower compared to that from unprotected rooms. The areas most contaminated with fungi were corridors and bathrooms. The dominant species were *Cladosporium* and *Penicillium*. *A. fumigatus* was rare in hematological departments (6/61, 9.8% examinations performed in 2014 and 2/40, 5% in 2019), whereas in the hospital for lung diseases an outbreak of *A. fumigatus* spores with up to 300 CFU/m^3^ was noted in March 2015. No triazole-resistant *A. fumigatus* isolate was detected. (4) Conclusions: Regular microbiological testing of the hospital environment can contribute to the detection of spore outbreaks, and thus enable the implementation of corrective procedures (e.g., additional disinfection, changing of HEPA filters).

## 1. Introduction

Fungi are regarded as one of the most widely spread and abundant group of biological air pollutants. The concentration and species composition of microorganisms present in the atmospheric air are constantly changing, depending on such factors as geographic location, seasons, climate (temperature, humidity), as well as rural or urban areas [1]. The changes are often cyclical in nature; in central Europe, the season of their most abundant occurrence is usually from May-June to September–November (depending on the year), and most of their spores appear during dry and windy days preceded by a warm, rainy period [1,2]. Increased humidity promotes the germination of spores and colonization of damp wall surfaces, bathroom fixtures, furniture, ventilation ducts and, in consequence, propagation through air. Outbreaks of mold spores, including *Aspergillus fumigatus*, can be observed during the demolition of old buildings or failures of ventilation systems [3]. The predominant molds outdoors are usually *Cladosporium* and *Alternaria*, whereas in premises they are *Penicillium* and *Aspergillus* genera, especially the species *P. chrysogenum, P. expansum, P. aurantiogriseum, A. versicolor, A. niger* and *A. flavus*. The next most frequent are representatives of the genera of *Cladosporium, Alternaria, Acremonium, Trichoderma, Rhizopus* and *Mucor* [1,4,5].

Fungal spores inhaled with air enter the paranasal sinuses and lower respiratory tract, where they can germinate, contributing to the development of respiratory disorders. The small size of the spores and airborne mycelial fragments (<1 µm) facilitates penetration into the lower respiratory tract. Fungal spores are equipped with numerous allergenic proteins (e.g., Alt a 1, present in *Alternaria* as well as in some other fungal genera); in addition, many molds produce mycotoxins, glucans and microbial volatile organic compounds [6]. Defense mechanisms in the human respiratory system, e.g., mucociliary clearance, mechanical barriers and cellular immunity (alveolar macrophages, neutrophils, airway epithelial cells), prevent the host from developing invasive fungal disease, unless these mechanisms are compromised by other pathogenic factors [7,8]. According to current estimates, the most common disease caused by airborne fungi is fungal asthma, which affects 10–25 million adults and ~2 million children globally [9,10]. *Alternaria* spp., *Cladosporium* spp. and *Aspergillus fumigatus* are considered the most allergenic; however, allergies can be caused by many other genera, e.g., *Mucor, Botrytis, Fusarium, Curvularia, Phoma* and *Rhizopus* [2,6]. The risk of respiratory tract infections is further limited by the fact that many environmental fungi are psychrophilic organisms that are incapable of growing at the body temperature of humans and warm-blooded animals. Among airborne pathogenic fungi, fungal disease can be caused in the first place by *Aspergillus fumigatus,* then other species of this genus (*A. flavus, A. terreus, A. niger*), some hylohyphomycetes, e.g., *Fusarium* spp., and representatives of *Mucormycetes*. The leading factor of *A. fumigatus* virulence is linked to its thermophilicity and ability to grow in a wide range of temperatures, as well as to the characteristics of its spores (small size, 2–3 µm, surface hydrophobins and a melanin layer that is responsible for the hydrophobicity and resistance to UV and oxidative stress). The persistence and multiplication of *Aspergillus fumigatus* in the respiratory tract occurs more easily in patients suffering from chronic pulmonary disorders, e.g., asthma, tuberculosis or sarcoidosis, and may lead to the development of allergic bronchopulmonary aspergillosis (ABPA) or different types of chronic pulmonary aspergillosis (CPA). In untreated cases, the 5-year mortality of CPA is estimated at 80% [11,12]. In addition, the emerging resistance or tolerance to triazole drugs can be a major problem in the therapy, and the prevalence of triazole resistance in patients with CPA and ABPA can reach 59% and 43%, respectively [13].

The most severe and life-threatening form of mycoses is considered an invasive fungal disease (IFD), and the most frequent is invasive aspergillosis (IA). The risk population includes patients with an impaired immune response, especially hematopoietic transplantation or solid organ transplantation recipients, acute leukemia patients and patients with inborn errors of immunity. Improved diagnostics of IFD has revealed multiple other risk groups, such as patients with exacerbated chronic obstructive pulmonary disease (COPD), who show invasive aspergillosis in up to 4% of cases [11]. Neutropenic patients with hematological malignancies and recipients of allogeneic hematopoietic stem cell transplantation (allo-HSCT) are still regarded as the population at greatest risk of IFD [10,14]. Due to neutropenia and a lack of phagocytosis, fungi that enter the respiratory system of these patients can invade the lung or sinuses, and hence spread to blood vessels and surroundings as well as to distant tissues and organs [11]. The development of this acute disease is usually rapid (<1 month), with a mortality rate in the range of 30–70%. To reduce IFD-related mortality, strategies of antifungal prophylaxis, empirical therapy and preemptive therapy (based on diagnostic screening) are used. However, prevention through the appropriate design of hospital wards to ensure the elimination, or at least a reduction in fungi in the indoor environment, is undoubtedly crucial. The risk can be reduced by the installation of air-conditioning systems with HEPA (high-efficiency particulate air) filters, a high frequency of air exchanges (at least 12 per hour) and appropriate differentiation of air pressure in individual rooms, limiting the influx of microorganisms from unprotected areas. The isolation rooms in such wards consist of a patient room with positive air pressure, a shower, a toilet, and an anteroom (with negative air pressure), which separates the patient room from the corridor (most often without a HEPA filter) [5,15]. The proper functioning of HEPA systems requires adequate technical supervision, including the replacement of filters, and should be supported with microbiological surveillance of the air quality [5].

The microbiological surveillance of fungal pathogens in high-risk environments involves routine air sampling, but there are no universal guidelines on the methodology. The sedimentation method is considered to be unsuitable for fungi; instead, a method using impactor samplers that determine the number of CFU/m^3^ air is most commonly used. According to one proposal, the limit that should not be exceeded in HEPA-filtered rooms is <0.1 CFU/m^3^ of *Aspergllus* spp., and 15 CFU/m^3^ of total fungi [5]. The isolation of pathogenic fungi from air samples is an indication to implement decontamination procedures, replace filters and, if necessary, inspect and repair the malfunctioning ventilation systems. The surveillance can be used for the detection and identification of drug-resistant isolates, which may affect antimicrobial stewardship.

The aim of this study was to investigate the fungal contamination of hospital environments in departments hospitalizing patients at high risk of IFD and, in particular, to evaluate the prevalence of *Aspergillus, Fusarium* and *Mucormycetes*.

## 2. Materials and Methods

### 2.1. Study Design

The study was conducted over two periods: from December 2014 to April 2015 (survey I), and from October 22 to 13 December, 2019 (survey II), and included mycological examination of the environment in selected hospitals in the same city (Wroclaw, Poland). Each time, the material for the study consisted of collecting indoor air samples from selected hospital rooms (patients rooms, bathrooms, treatment rooms, corridors); additionally, during survey II, environmental swabs taken from bathroom surfaces were also tested. Survey I involved different facilities of three hospital wards: hematology (HEM I), pediatric hematology (HEM II), pulmonology in hospitals for lung diseases (LDH), each of which was located in Wroclaw, in different buildings belonging to the so-called “old buildings”. The age of the oldest building was 123 years (LDH), and the youngest was 50 years (HEM II). Survey II included three hospital units, of which one pulmonology (LDH) unit was located in a building that was built in 1892 (the same, which was tested in survey I), whereas the other two were pediatric hematology units (transplant and post-transplant) in a new building that was put into use in 2015. This building had a central ventilation system with HEPA filters installed in some rooms (details provided in the “Results”). All of the old buildings (HEM I, HEM IIB, HEM IIC, LDH) were equipped with natural (gravity) ventilation systems, and only the patient rooms of the bone marrow transplant units (HEM IIA) had mechanical ventilation systems equipped with HEPA filters. In patient rooms of the HEM I building we studied, there were additionally set up free-standing devices [GENANO310 Medical Air Cleaning System; Genano Ltd., Espoo, Finland] that disinfected the air, capturing and eliminating microorganisms based on patented non-thermal plasma technology. All of the facilities were operating as hospitals since their beginning. Several cases of invasive fungal diseases were diagnosed annually in the hematology departments. The study conducted was not related to any epidemic outbreak or increase in the number of observed fungal infections. The surveys were not a part of a plan for routine environmental inspections; however, each time, samples were taken in consultation with and under the control of the relevant Hospital Infection Control Committee, with which the results were shared.

### 2.2. Air Examination

Microbiological analyses of the air were carried out with the use of the MicroBio MB1 air sampler (Cantium Scientific, Clarendon Gardens, UK), according to the manufacturer’s instructions. Open 90 mm petri dish plates with Sabouraud dextrose agar supplemented with chloramphenicol (BioMaxima, Lublin, Poland) were placed in the apparatus, and a perforated metal head (220 holes with a diameter of 1 mm) was applied. The apparatus was programmed to draw a fixed amount of air (150–500 L) at a flow rate of 100 L/min. From each room, 3–5 air samples were taken. After sampling, the plates were placed in sterile bags and transported to the laboratory within an hour. The sampling times were adapted to the work of the department, usually between 12 pm and 3 pm. The medical staff and patients were asked not to open windows on the day of sampling (in rooms with tilt-up windows). During the measurements, the windows and doors in the tested rooms were closed, and the air sampler was about 1.2–1.4 m above the ground. Before each day of sampling, the head was sterilized in an autoclave, while during measurements between rooms, the instrument head was disinfected with Aerodesin 200 (Medilab, Białystok, Poland). The plates were incubated at 25 °C for 2–10 days, and fungal growth was observed every 48 h. The number of grown colonies was counted each time, in which the fungal growth sites on the plate were marked with a marker to avoid counting secondary colonies. Based on the number of colonies grown on each plate, the value of CFU (colony forming unit) in 1 m^3^ of air was calculated using the following formula:

$$\mathrm{CFU}/m^{3}=1000\times \frac{n_{c}}{V_{s}}$$

(n_c_—corrected number of colonies counted; V_s_—volume of sampled air in liters).

The corrected colony count (n_c_) was read from the appendix added to the MicroBio MB1 instrument manual. The final result was the arithmetic mean obtained from three measurements.

Swabs were taken from the grout of shower trays and sinks, selecting areas with an altered colour, suggesting the presence of fungi. The swabs were taken using a sterile swab that was moistened with sterile saline solution (0.9% NaCl), by rubbing it and rotating it around its axis on the surface to be examined. The swabs were transported to the laboratory within one hour and subsequently, the material collected on each swab was spread over the entire surface of each Sabouraud agar plate. The plates were incubated at 25 °C for 2–10 days, as described above.

### 2.3. Microbiological Proceeding

The cultured molds were identified on the basis of morphological (colony morphology, microscopic preparations in lactophenol) and physiological features (the ability to grow at 37 and 40 °C) to the level of genera and/or species complexes. The morphology of selected strains after subculture on Czapek-Dox and malt extract agars (BioMaxima, Poland) were also evaluated. Based on these preparations, the fungi were classified as *Aspergillus fumigatus* species complex, *Aspergillus* spp. (other than *A. fumigatus*), *Penicillium, Cladosporium, Alternaria, Fusarium* and *Mucormycetes*. The remaining cultured molds, that could not be classified into the above-mentioned taxa, were described as “other molds”. Selected isolates of the yeast-like fungi were identified with the use of BD Phoenix™ YEAST ID panels, according to the manufacturer’s instructions; however, as it was not possible to identify all of the yeast colonies using this method, they were described on the basis of their growth morphology as “yeast-like fungi”, with/without orange pigmentation.

### 2.4. Susceptibility to Triazole Derivatives

The isolates that were identified as *Aspergillus fumigatus* species complex were screened for their susceptibility to itraconazole, posaconazole and itraconazole, using the microdilution method according to EUCAST [16]. Serial dilutions (0.03–32 mg/L) of the tested antifungals were prepared in the medium RPMI1640 2× buffered with MOPS and dispersed in 100 µL aliquots on sterile 96-well microplates. To prepare inoculum spores from 2–3 day old cultures of *Aspergillus* on Sabouraud agar, the slants were suspended in sterile distilled water supplemented with 0.1% Tween 20 to obtain a density of McFarland 0.5. After dilution 10 times, suspensions of densities 1–2.5 × 10^5^ CFU/mL were dispersed on the microplates with antimycotics (100 µL per well). The plates were incubated for 48 hrs at 35 °C, and the MIC was read visually as the lowest drug concentration that resulted in 100% inhibition of fungal growth. The results were interpreted according to EUCAST breakpoint tables for interpretation of the MICs [17]. The strains with MIC values of >1 mg/L for ITR and VOR, and >0.25 mg/L for POS, were regarded as resistant, while values of ≤1 mg/L for ITR, VOR, and ≤0.125 mg/L for POS were regarded as susceptible. The antimycotics and reagents used (DMSO, RPMI 1640 medium, MOPS buffer) were obtained from Sigma-Aldrich.

### 2.5. Statistical Analysis

The fungal counts were analyzed with the use of Student’s t and U Mann–Whitney tests and Statistica 13.1 1 (TIBCO, Software Inc. 2017, STATISTICA, version 13, Dell, OK, USA) software.

## 3. Results

### 3.1. Air Examination

#### 3.1.1. Hematology Wards

Mycological air contamination in the hematology ward (HEM I) was investigated in December 2014 and April 2015. The study included 6 one- or two-bed patient rooms equipped with devices for air disinfection (described in “Methods”), as well as 10 other rooms, listed in Table 1. The total number of fungi per cubic meter of the air was higher in December than in April, in both in patient rooms (13–43 versus 0–7 CFU/m^3^) as well as in the rest premises under study (up to 87 in December vs. up to 20 CFU/m^3^ in April). The pathogenic *Aspergillus fumigatus* species was detected in 2 out of 3 corridors tested in December 2014 (13 CFU/m^3^), whereas *Aspergillus* spp. were detected in December and in April in patients’ bathrooms (3–13 CFU/m^3^), nurses’ rooms (3–10 CFU/m^3^) and corridors (0–7 CFU/m^3^) (Table 1).

**Figure 1:**
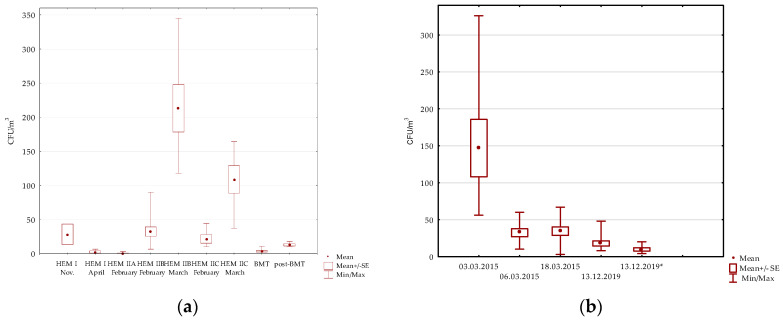
Concentrations of fungi (CFU/m^3^) in the air of patient rooms in hematological wards (**a**) and in patient rooms and bathrooms (13.12.2019; marked with an asterisk) in hospitals for lung diseases (**b**). The graphs show mean values of CFU/m^3^ as well as standard errors (SE) and minimal and maximal values obtained for particular hospital wards listed in Table 1 and Table 2.

**Figure 2:**
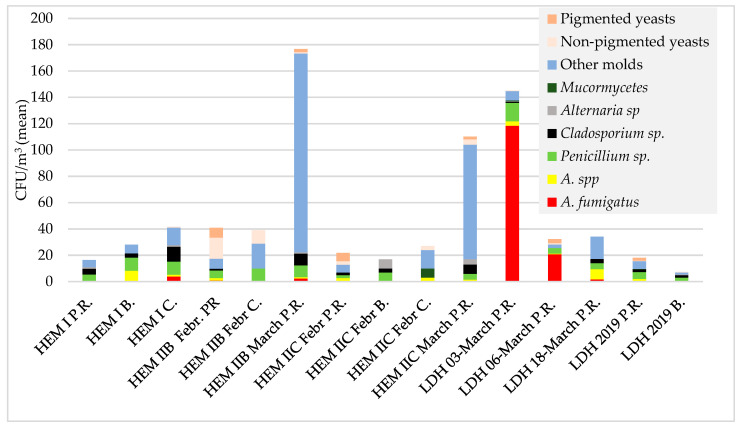
Species distribution in air samples from hematology units (HEM I, HEM II B, HEM IIC) and hospitals for lung diseases (LDH) wards. The graph shows mean CFU/m^3^ values for each fungal species/morphotype. For HEM I ward, the results obtained in December 2014 and April 2015 are summarized together; for HEM IIB and HEM IIC, the results obtained in February 2015 and March 2015 are presented separately. The following abbreviations are used: P.R.—patient rooms, B.—bathrooms, C.—corridors. The term “other molds” includes hyalohyphomycetous fungi, different from the other listed genera. Among “other molds” isolated in March from HEM IIB and HEM IIC, *Chrysonilia* predominated; the remaining molds were not identified to the genus level.

The pediatric hematology (HEM II) units studied in survey I were divided into three zones. The patient rooms in zone A were equipped with HEPA filters and positive air pressure, whereas those in zones B and C were not. Section A (8 patient rooms, kitchen, nurses’ room, corridor) was tested only in February 2015, and sections B and C (11 and 7 patient rooms, respectively, and adjacent premises) were tested two times in a one-month interval. No *Aspergillus* fungi were detected in any air samples taken from zone A. Fungi were detected only in 1/8 of the patient rooms (3 CFU/m^3^) and in the nurses’ station (3 CFU/m^3^) (Table 1). In the case of sections B and C, the results were the opposite; fungi were detected in air samples from all of the tested rooms in February, as well as in March. In February, the number of fungi per cubic meter in rooms of zone B ranged from 7 to 90 CFU/m^3^ (mean 30 CFU/m^3^), with *Aspergillus fumigatus* found in 2/11 of the patient rooms (18%) (3–7 CFU/m^3^) and *Aspergillus* spp in the next 3/11 (27%) (7 CFU/m^3^) patient rooms. The most common microorganisms were *Penicillium* spp. (72% rooms) and yeasts (54%). In March, the number of fungi in the air ranged between 118–345 CFU/m^3^ (mean 213 CFU/m^3^; *p* < 0.001, comparing to February). *Aspergillus fumigatus* was present in 3/7 (43%) rooms, while the most prevalent were *Cladosporium* spp. (100% rooms), *Penicillium* spp. (85%) and “other molds”, of which *Chrysonilia* spp. dominated (100%). Similarly, in rooms belonging to section C (7 patient rooms, bathrooms, treatment rooms and the corridor) the total number of fungi in the air samples taken in February was lower than in March (10–44 CFU/m^3^; mean 20 CFU/m^3^ versus 37–164 CFU/m^3^; mean 108 CFU/m^3^; *p* < 0.005). No *Aspergillus fumigatus* was detected, while *Aspergillus* spp. were present in 4/7 (57%) of the patient rooms and in the corridor. In addition, a *Mucor* spp. culture (7 CFU/m^3^) was obtained from the corridor (Table 1, Figure 1 and Figure 2).

In autumn 2015, the pediatric hematology unit was moved to a new building that was located in another part of the city, and was the subject of survey II, which was performed in October and November 2019. Due to the outbreak of the COVID-19 pandemic, the study could not be continued in spring 2020 as originally planned. The examination was conducted in the transplant (BMT) and post-transplant (post-BMT) wards. In the BMT ward, 8 patient rooms (isolation rooms) with their adjacent sluices (anterooms) and bathrooms, as well as the corridor leading to them, were surveyed. For organizational reasons, the samples from isolation rooms, sluices, and the corridor were taken on 22.10.2019, while from bathrooms the samples were taken on 12.11.2019. The building possessed a central ventilation system, with HEPA filters installed in the patient rooms (both BMT and post-BMT). Additionally, patient rooms of the BMT area were protected with positive air pressure and negative pressure anterooms.

The presence of fungi was detected in 23/24 (95.8%) of the rooms surveyed in the BMT. Contamination of the indoor environment was lowest in the patient rooms, and ranged from 0 to 11 CFU/m^3^; in the sluices leading to the rooms, it was 2–36 CFU/m^3^ (*p* < 0.05); and in the bathrooms, the contamination was 2–32 CFU/m^3^. The air samples were found to be the most contaminated in the corridor, as in that area the calculated value was 237 CFU/m^3^. The mold most frequently identified, in 15 of the 24 rooms, was *Cladosporium* spp., and their average values in the patient rooms, bathrooms and sluices reached 3.5 CFU/m^3^, 8.75 CFU/m^3^ and 10 CFU/m^3^, respectively. The genus was most abundant in the corridor, where its value reached 131 CFU/m^3^. The next most frequently identified mold were *Penicillium* spp., which were cultured in samples from 4 rooms (16.7%). In addition to the mentioned genera, *Fusarium* spp. and *Aspergillus fumigatus* were detected in the corridor and in one of the bathrooms. In addition to molds, yeast-like fungi were detected in 8/24 (33%) rooms. Most of the isolated yeasts (6/8) were orange-pigmented (most probably belonging to *Rhodotorula* spp.). Detailed results are shown in Table 1 and Figure 1 and Figure 2. *Fusarium* sp. and orange-coloured yeast were abundant in swabs that were taken from the grout in shower trays and sinks in patients’ bathrooms.

The post-BMT unit was sampled on 12 November 2019. The survey covered five single-bed patient rooms with bathrooms adjacent to these rooms, as well as the corridor between the rooms, and the nurses’ counter. In addition, the air in corridor II, leading to BMT and post-BMT units and the two elevators in it, was also surveyed.

**Figure 3:**
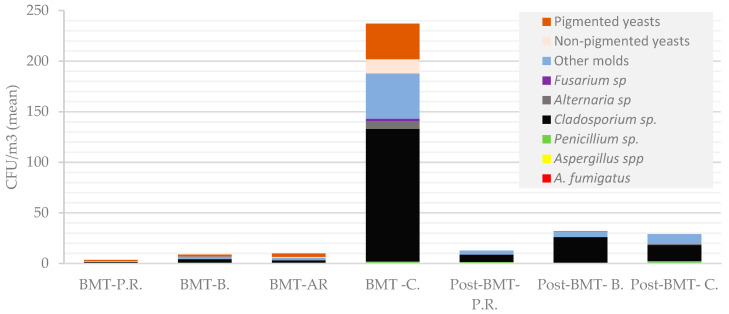
Concentrations of particular fungal species/morphotypes (CFU/m^3^) in the air of different rooms of BMT and post-BMT wards. The following abbreviations were used: P.R.—patient rooms, AR—anteroom, B.—bathrooms, C.—corridors.

Fungi were cultured from all of the rooms examined, and the total number of fungi per cubic meter ranged from 6–54 CFU/m^3^, with 10–18 (mean 12.8) CFU/m^3^ in patient rooms, and 6–28 (mean 19.6) CFU/m^3^ in bathrooms. In all of the rooms, the genus *Cladosporium* was present, in 8/15 (53%) of them, *Penicillium* spp. was also recognized. No *Aspergillus* spp., *Fusarium* spp., or *Mucormycetes* were detected in any of the rooms (Table 1; Figure 3).

##### Pulmonology Hospital

During survey I, air samples from rooms in the hospital for lung diseases were taken in March 2015 (3 March 2015, 6 March 2015, and 18 March 2015). The study included 1 operating theatre room and 22 patient rooms (2 of them surveyed twice). Molds were detected in all of the air samples, with the number of CFU per 1 m^3^ of air ranging from 10 to 325.56 CFU (mean 69.37 CFU/m^3^). The presence of *Aspergillus fumigatus* species was recorded in 20/25 (80%) of the samples, with CFU/m^3^ values ranging from 3 to 307.8, and *Aspergillus* spp. were present in 5/25 (20%) of the samples. The other fungi that were isolated were *Penicillium spp.* and *Cladosporium spp* present in 18/25 (72%) and 6/25 (24%) of the samples, respectively, as well as *Mucor* spp. and *Alternaria* spp. identified in 2 (8%) rooms. The presence of yeasts was noted in 4/25 (16%) rooms (Table 2, Figure 1b and Figure 2).

The material for survey II was collected on 13 December 2019. The study included 10 patient rooms (6 singles and 4 triples), bathrooms (4), the nurse’s room, and the operating theatre. The fungi from each room were cultured, and their total value per cubic meter in patient rooms ranged from 8–48 (mean 18) CFU/m^3^, and in the rest of the rooms it was 4–20 (mean 10) CFU/m^3^. The genus *Aspergillus* was present in samples from 7/10 (70%) of the patient rooms, including one positive for *Aspergillus fumigatus.* The most frequently isolated molds were *Penicillium* spp., detected in 9/10 (90%) of the patient rooms and in 3/4 (75%) bathrooms. Other mould genera identified included *Cladosporium* (7/10 patient rooms), *Rhizomucor* (1/10 patient rooms) and *Alternaria* found in the air in 1/4 of the bathrooms. Positive yeast culture results were obtained in samples from 7/10 (70%) patient rooms, from which two yeasts without pigment were cultured (Table 2; Figure 1). In the bathrooms, additional mycological tests of swabs taken from the grout of sinks and shower trays were performed. The cultures of swabs from all of the bathrooms revealed the presence of *Fusarium* spp. and orange-pigmented yeast-like fungi.

### 3.2. Aspergillus Fumigatus Species Complex Susceptibility to Triazole Derivatives

Thirty-one isolates of *Aspergillus fumigatus* species complex obtained during survey I were examined for their susceptibility to three triazole derivatives: itraconazole, voriconazole and posaconazole (Table 3). All of the isolates were classified as susceptible to tested drugs, according to clinical breakpoints established by the European Committee on Antimicrobial Susceptibility Testing [17]. The minimal concentrations inhibiting 50 (MIC_50_) and 90 percent of tested isolates (MIC_90_) were 0.25 and 1 for itraconazole, 0.25 and 0.25 for voriconazole, and 0.06 and 0.06 for posaconazole, respectively.

## 4. Discussion

The role of environmental surveillance is less proven in fungal than in bacterial infections. The high intraspecies variability of fungal pathogens impedes the discovery of associations between environment colonization and the etiology of IFD [18]. A recent study describing an investigation of the sources of fungal infection in patients with COVID-associated aspergillosis failed to demonstrate a link between clinical strains and those isolated from hospital environments (no shared genotypes) [19]. A similar French study conducted on more than 200 clinical and environmental *A. fumigatus* isolates showed the importance of integrated studies, as despite the high genotypic variability, it was not possible to exclude hospital transmission in some of the analyzed cases [20]. Routine testing of the hospital environment tends to focus on bacteria, and the search for fungi is rarely requested, mainly due to the limited access to reference mycological laboratories, the high cost, and difficulties in results interpretation.

The age of a building affects the degree of microbial colonization of its indoor environment. The first phase of our study (2014/2015) involved rooms in three different old buildings, while the second phase (2019) involved one new building and one old one. The examinations performed both in the old and in new buildings’ hematology departments revealed that air in the rooms equipped with sterile air circulation (HEPA filters) contained minimal contamination with fungal pathogens. The concentration of fungi was low (up to 11 CFU/m^3^), and no *Aspergillus* was cultured, meeting the requirements suggested for heavily protected areas [5,21]. Interestingly, a comparison of the number of fungi in the isolation rooms of the BMT and patient rooms of the post-BMT showed no statistical differences, even though there was positive air pressure in the BMT in addition to the HEPA filters. It can be hypothesized that the very good result in April 2015 in the hematology department (HEM I) may have been affected by the use of an air-flow disinfection device. The device (Genano 310 Medical Air Cleaning System; Genano Ltd., Finland) was first installed in September 2007. As shown in an earlier study, the baseline results of 380 CFU/m^3^ and 200 CFU/m^3^ for *Aspergillus* spp. decreased significantly (45 CFU/m^3^ and 0–5 CFU/m^3^ of *Aspergillus*) after installation of the system, but seasonal fluctuations were observed [22]. The seasonal fluctuations may have been affected by other environmental factors (opening of windows, doors) that caused an influx of fungal spores and compromised the efficacy of air-flow disinfection. In designing the study, we primarily wanted to determine whether pathogenic fungi were present in the indoor air at the time of the study, and thus assess the risk to patients and/or medical staff. At this point, it should be noted that due to the natural type of ventilation, a significant part of the cultured microorganisms came from the outside. Conducting a simultaneous study of the concentration of fungal spores in the outdoor air would have been helpful in clarifying the external/internal origins of the cultured fungi. The highest air contamination and fungal colony density was observed in the bathrooms, even in the modern BMT unit, and the fungal flora contained potentially pathogenic species such as *A. fumigatus* and *Fusarium* spp. *Fusarium* spp. and *Rhodotorula* spp. were also detected on the surface of the grout in the shower trays and washbasins. This observation is in line with reports from other authors, who also identified bathrooms as a likely source of fungi in protected hematology wards, with cultured *Aspergillus* spp. found in almost a quarter of them [23].

Universally, in all of the buildings that were studied, the highest concentration and the greatest diversity of fungi were observed in corridors. This also applied to corridors in the new building, in which mechanical ventilation existed, but without HEPA filters. Reboux et al., during their 10-year surveillance, observed several cases of increased *Aspergillus* concentrations in hospital corridors (40 CFU/m^3^), and suggested that this may indicate an increased risk of invasive fungal infections for patients [24].

The varying levels of air contamination in hematology units was also illustrated by a study by Tığlı et al. [25] conducted in Turkey. Eleven rooms, both transplant and general wards, were included in the study, and the total number of colony-forming units per cubic meter ranged from 0 to 34 CFU and 0 to 797 CFU, respectively, including *Aspergillus* reaching levels of 0 to 3 CFU/m^3^ and 0–35 CFU/m^3^, respectively. Despite the distinct climate, the values obtained by Tığlı et al. in rooms with controlled air circulation were similar to the results obtained in the study presented here, while the values obtained in the general ward were higher. In the present study, non-protected hematology patient rooms were investigated in February and in March 2015 (HEM IIB and HEM IIC), with contamination reaching levels of up to 44 and 345 CFU/m^3^ in general fungal load, respectively, and *Aspergillus* spp. contamination ranging from 0–7 CFU/m^3^.

A similar degree of fungal contamination was noted in unprotected rooms of the pulmonary hospital, with one exception. The air samples taken on 3 March 2015 were heavily contaminated with *Aspergillus fumigatus* spores, the number of which exceeded 300 CFU/m^3^ in some patient rooms (Table 2, Figure 1b and Figure 2). The alarming results of the first cultures and the dominance of *A. fumigatus* prompted the use of vaporized hydrogen peroxide decontamination. The control examination, performed after this process (18.03.2015), showed a strong reduction in the occurrence of *A. fumigatus* and *Aspergillus* spp. spores; however, they were not completely eliminated. In the next survey in December 2019, the overall fungal number was 48 CFU/m^3^, including up to 4 CFU/m^3^ of *Aspergillus* spp. The reason for such a high number of *A. fumigatus* in March 2015 was not established. The hospital was located near green areas and residential buildings, which are often remodeled. It is possible that renovation work, carried out in neighboring buildings or related to the care of greenery, emitted a cloud of spores, some of which reached the hospital site. Certainly other factors, such as the lack of modern ventilation systems or the age of the hospital building (120 years), cannot be ruled out. Unfortunately, we were not able to test for the concentration of fungi in the outdoor air, which may have helped determine the source of spore emissions. A similar situation, described as a temporary deviation from the norm in the number of *Aspergillus* spp. in air samples, was reported by the team of Falvey and Streifer in their study conducted at the University Hospital of Minnesota. During their research over a period of 10 years, the researchers observed this phenomenon several times [26]. Hospital renovation/construction is considered to be a major source of invasive healthcare-associated fungal infections; thus, planning such activities requires the preparation of appropriate safeguards [5,15,21]. It can be emphasized that there is a need for monitoring airborne fungal spore concentrations and determining breakpoint values [27]. Establishing comparable methods of air sampling and fungal spore detection was recommended by different sources. According to the CDC, the volume of aspirated air in a highly protected area should be at least 1000 L, and the examination should be limited to fungi growing at 35 °C [21]. In our study, 500 L of air was aspirated on agar plates and incubation was carried out at 28 °C. The use of higher temperatures reduces the growth of many environmental fungi, such as *Cladosporium* spp., or *Alternaria* spp., which are not the cause of invasive infections, but their presence is also not indifferent to human health. A very important limitation of our study was the lack of molecular testing, which enables identification to the species level, including so-called cryptic species that are indistinguishable by classical methods. The high proportion of molds isolated from old buildings was not identified even to the genus level, due to a lack of typical morphological structures.

Due to increasing reports of *A. fumigatus* resistance to triazoles, isolates of *A. fumigatus* were tested against the most commonly used triazole drugs (itraconazole, voriconazole and posaconazole). All of the strains tested were found to be sensitive to these drugs. This is in agreement with our previous studies on clinical isolates, among which resistance to azoles was detected very rarely in our region (0–4%) [28,29].

The microbiological control of air in healthcare facilities remains a challenge. The research presented here is one of many examples that, despite there being many problems and unresolved questions (e.g., data interpretation), the examination of the hospital environment provides important information that can be used to improve the safety of patients at risk. The study examined air samples collected on selected dates and in randomly selected rooms. The results obtained, especially for the detection of the presence of potentially pathogenic species, indicate the need for periodic microbiological (mycological) surveillance of the hospital environment. Comparative studies of outdoor air pollution should also be considered.

## Figures and Tables

**Table 1:** Concentrations of fungi in the air of hematological departments.

Hospital Ward	Data	Type of Room (Number of Rooms Tested)	Cultured Fungi [CFU/m^3^] (Number of Positive Rooms/Number of Rooms Tested)									
			Total Number of Fungi Range (Mean)	*A. fumigatus*	*Aspergillus* spp.	*Penicillium* sp.	*Cladosporium sp.*	*Alternaria* sp	*Mucormycetes*	Other Molds	Non-Pigmented Yeasts	Pigmented Yeasts
HEM I	11.12.2014	Patient rooms (2)	13.33–43.33	-	-	-	0–26.6	-	-	13.3–16.3	-	-
		Patient’s bathroom (1)	36.67	-	13.33	3.33	6.6	-	-	13.3	-	-
		Corridors (3)	50–86.7 (64.4)	0–13.3 (2/3)	0–6.6 (1/3)	13.4–16.6 (3/3)	6.6–30 (3/3)	0–6.6 (1/3)	-	6.6–36.6 (3/3)	0–3.3 (1/3)	*-*
		Nurses’ station (1)	56	0	10	15	-	-	-	30	-	-
	14.04.2015	Patient rooms (4)	0–6.67 (2.5) (2/4)	-	-	3.3 (1/4)	-	0–6.6 (1/4)	-	0–3.3 (1/4)	-	*-*
		Patient’s bathroom (1)	20	-	3.33	16.6	-	-	-	-	-	-
		Treatment room (1)	3.33	-	-	-	-	-	-	3.3	-	-
		Nurses’ station (1)	23.33	-	3.33	3.33	6.6	-	-	6.6	3.33	*-*
		Corridor (2)	3.33–13.33	-	-	0–6.6	3.3–3.3	-	-	0–3.3	-	-
HEM II (part A)	6.02.2015	Patient rooms (8)	0–3.33 (1/8)	-	-	-	-	-	-	0–3.33 (1/8)	-	-
		Nurses’ station (1)	3.33	-	-	3.33	-	-	-	-	-	-
		Corridor (1)	0	-	-	-	-	-	-	-	-	-
		Kitchen (1)	0	-	-	-	-	-	-	-	-	-
HEM II (part B)	10.02.2015	Patient’s room (11)	6.6–9 (30) (11/11)	0–6.67 (2/11)	0–6.6 (3/11)	0–16.6 (8/11)	0–6.6 (3/11)	-	-	0–13.3 (10/11)	0–30 (6/11)	0–26.6 (6/11)
		Corridor (1)	40	-	-	10	-	-	-	19	10	-
	26.03.2015	Patient rooms (7)	118–345 (213) (7/7)	0–7 (3/7)	0–7 (1/7)	0–20 (6/7)	3–20 (7/7)	0–7 (1/7)	-	83–288 (151) (7/7)	0–7 (1/7)	0–17 (1/7)
HEM II (part C)	20.02.2015	Patient rooms (5)	10–44 (20) (5/5)	-	0–7 (2/5)	0–5 (3/5)	0–7 (2/5)	-	-	0–1 (3/5)	0–14 (1/5)	0–24 (2/5)
		Patient’s bathroom (1)	17	-	-	7	3	7	-	-	-	-
		Treatment room (1)	7	-	-	-	-	-	-	7	-	-
		Corridor (1)	27	-	3	-	-	-	7	14	3	-
	26.03.2015	Patient rooms (6)	37–164 (108)	-	0–7 (2/6)	0–17 (4/6)	0–27 (4/6)	0–10 (3/6)	-	24–153 (87)	0–10 (4/6)	0–7 (3/6)
BMT	22.10.2019	Patient isolation rooms (8)	0–11 (3.5)	-	-	-	0–4 (5/8)	0–1 (1/8)	-	0–1 (2/8)	0–1 (1/8)	0–8 (2/8)
		Sluices (8)	2–36 (10)	-	-	0–4 (2/8)	0–10 (3/8)	0–2 (1/8)	-	0–14 (2/8)	0–6 (1/8)	0–18 (2/8)
		Corridor (1)	237	1	-	1	131	8	-	47 ^F^	14	35
		Bathrooms (8)	2–32 (8)	0–2 (1/8)	-	0–4 (1/8)	0–14 (7/8)	-	-	0–6 ^F^ (5/8)	-	0–12 (2/8)
Post-BMT	12.11.2019	Patient rooms (5)	10–18 (12.8)	-	-	0–4 (3/5)	2–12; (5/5)	-	-	2–8 (5/5)	-	-
		Bathrooms (5)	6–28 (19.6)	-	-	0–2 (1/5)	4–22; (5/5)	-	-	2–12 (5/5)	-	0–2 (1/5)
		Corridor (1)	54	-	-	4	15	2	-	32	1	-
		Nurses’ counter	18	-	-	4	2	-	-	12	-	-
Communication routes BMT/post-BMT	12.11.2019	Elevator I	36			2	28			2		
		Elevator II	20			4	16					
		Corridor by the elevators	10				4			6		

^F^—*Fusarium* spp. included (2 CFU/m^3^).

**Table 2:** Concentrations of fungi in the air of the hospital for lung diseases.

Data	Type of Room (Number of Rooms Tested)	Total Number of Fungi [CFU/m^3^] Range (Mean)	Cultured Fungi [CFU/m^3^] (Number of Positive Rooms/Number of Rooms Tested)								
			*A. fumigatus*	*A.* spp.	*Penicillium* sp.	*Cladosporium* sp.,	*Alternaria* sp.	*Mucor mycetes*	Other Molds	Non-Pigmented Yeasts	Pigmented yeasts
3.03.2015	Patient rooms (8)	56–326 (146.8)	45–300 (118.5 ^) (8/8)	0–17 (4/8)	3–26 (14.1^) (8/8)	0–6 (1/8)	-	0–3 (2/8)	0–32 (7/8)	0–3 (1/8)	-
6.03.2015	Patient rooms (8)	10–60 (32.3)	7–37 (20.8 ^) (8/8)	0–3 (1/8)	0–17 (6/8)	-	-	-	0–7 (5/8)	0–7 (1/8)	0–17 (2/8)
18.03.2015	Patient rooms (8)	3–67 (34.4)	0–7 (4/8)	0–23 (4/8)	0–13 (5/8)	0–10 (4/8)	-	-	0–33 (7/8)	-	-
	Treatment room	40	0	3	0	10			30		
13.12.2019	Patient rooms (10)	8–48 (18)	0–4 (1/10)	0–4 (6/10)	0–28 (9/10)	0–8 (5/10)	-	0–2 (1/10)	2–16 (6 ^) (10/10)	0–2 (2/2)	0–6 (5/10)
	Bathrooms (4)	4–10 (8)			0–6 (3/4)	0–4 (3/4)	0–2 (1/4)		0–2 (3/4)	0–2 (1/4)	
	Treatment room	7			2				2	1	
	Nurses’ room	20			6	2			14		

^—a mean value of CFU/m^3^.

**Table 3:** Minimal inhibitory concentrations (mg/L) of itraconazole, voriconazole and posaconazole against isolates of *Aspergillus fumigatus* species complex.

Hospital Ward	Number of Isolates (n = 31)	MIC (mg/L)		
		Itraconazole	Voriconazole	Posaconazole
Lung Diseases (24)	(17)	0.25	0.25	0.06
	(6)	1	0.25	0.06
	(1)	0.5	0.25	0.06
HEM I (1)	(1)	0.5	0.25	0.03
HEM II part B (5)	(4)	0.25	0.25	0.06
	(1)	1	0.5	0.06
HEM II part C (1)	(1)	1	1	0.125
MIC_50_	(31)	0.25	0.25	0.06
MIC_90_	(31)	1	0.25	0.06

## Data Availability

Not applicable.
